# Unexpected Role of pH and Microenvironment on the Antioxidant and Synergistic Activity of Resveratrol in Model Micellar and Liposomal Systems

**DOI:** 10.1021/acs.joc.1c01801

**Published:** 2021-11-29

**Authors:** Adrian Konopko, Grzegorz Litwinienko

**Affiliations:** † Faculty of Chemistry, University of Warsaw, Pasteura 1, Warsaw 02-093, Poland; ‡ Nencki Institute of Experimental Biology, Polish Academy of Sciences, 3 Pasteur Street, Warsaw 02-093, Poland

## Abstract

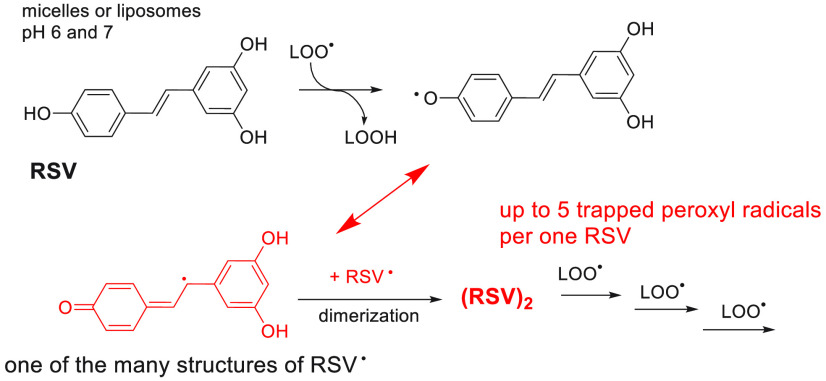

Experimental and theoretical studies indicate that resveratrol (RSV, dietary polyphenol that effectively reduces cellular oxidative stress) is a good scavenger of hydroxyl, alkoxyl, and peroxyl radicals in homogeneous systems. However, the role of RSV as a chain-breaking antioxidant is still questioned. Here, we describe pH dependent effectiveness of RSV as an inhibitor of peroxidation of methyl linoleate in Triton X-100 micelles and in 1,2-dimyristoyl-*sn*-glycero-3-phosphocholine (DMPC) liposomes, with the best effectiveness at pH 6 (stoichiometric factors, *n*, are 4.9 and 5.6, and the rate constants for reaction with peroxyl radicals, *k*_inh_, are 1200 and 3300 M^–1^ s^–1^ in micellar and liposomal systems, respectively). We propose the mechanism in which RSV-derived radicals are coupled to dimers with recovered ability to trap lipidperoxyl radicals. The formation of such dimers is facilitated due to increased local concentration of RSV at the lipid–water interface. Good synergy of RSV with α-tocopherol analogue in micelles and liposomes is in contrast to the previously reported lack of synergy in non-polar solvents; however, the increased persistency of tocopheroxyl radicals in dispersed lipid/water systems and proximal localization of both antioxidants greatly facilitate the possible recovery of α-TOH by RSV.

## Introduction

Among thousands of phytochemicals exhibiting antioxidant activity *in vitro* and *in vivo*, great attention has been paid to polyphenols.^1^ During the last 20 years, resveratrol (3,4′,5-trihydroxy-*trans*-stilbene, RSV) has gained particular interest because this molecule efficiently protects against environmental stress and pathogenic attack not only in plants but also in mammal organisms.^2^ The growing interest started from the 1990s, when a cardioprotective effect of consumption of RSV present in red grapes and red wine was discovered, but RSV can be found in many other plant species.^3^ A number of *in vitro* and *in vivo* studies proved that RSV exhibits therapeutic potential.^4^ Health benefits include cardioprotective and anti-inflammation effects, prevention against Alzheimer’s disease and several liver diseases, and decrease of blood glucose level and plasma lipids in mice with a diabetic disorder.^5^ Moreover, there are a number of reports on the anticancer activity of RSV^6^ against several types of cancers: leukemia, hepatocellular, or prostate carcinoma.^7^ Cardioprotective effects of RSV are connected with reduction of oxidative stress not only due to indirect antioxidant action (RSV upregulates expression of glutathione peroxidase, catalase, and heme oxygenase-1) but also due to a direct decomposition of H_2_O_2_.^8^ Intensive clinical and biochemical research was followed by a number of studies of the direct antiradical and antioxidant activity of RSV. Experimental studies^9^ and theoretical calculations^10^ resulted in the general conclusion that H atom abstraction (realized via a one-step or multistep process) produces a relatively persistent radical.^9e,10c^ A number of studies employed artificial model radicals like 2,2-diphenyl-1-picrylhydrazylradical radical (dpph^•^), ABTS (2,2′-azino-bis(3-ethylbenzothiazoline-6-sulfonic)) radical cation, and galvinoxyl radical. In methanol, the reaction of dpph^•^ with RSV is 6-fold slower than that with Trolox (water-soluble analogue of α-tocopherol, the most active natural antioxidant).^11^ The reaction with dpph^•^ in polar solvents proceeds via mixed mechanisms with hydrogen atom transfer (HAT) and sequential proton loss electron transfer (SPLET).^12^ Reactions with strongly oxidizing radicals like hydroxyl,^9b,9c,13^ alkoxyl,^14^ and chloroperoxyl^13,15^ are much faster—experimentally determined rate constants in water or water/organic solvents are within the range 10^7^–10^10^ M^–1^ s^–1^ and are pH dependent.^13^ Reaction of RSV with the less reactive hydroperoxyl radical (*k*_HOO^•^_ = 1.42 × 10^5^ M^–1^ s^–1^ in the aqueous phase) was interpreted as being exclusively due to phenolic hydrogen abstraction.^10f^ However, recently, Cordova-Gomez et al.^10e^ calculated *k*_HOO^•^_ = 5.01 × 10^4^ M^–1^ s^–1^ for neutral RSV, while deprotonated RSV reacts much faster, with a rate constant as high as 4.4 × 10^9^ M^–1^ s^–1^. Combination of those two rate constants with the concentrations of the corresponding molar fractions of ionized (1.7%) and neutral (98.3%) RSV at pH 7.4 gave the overall *k* = 5.6 × 10^7^ M^–1^ s^–1^.^10e^

**Scheme 1:**
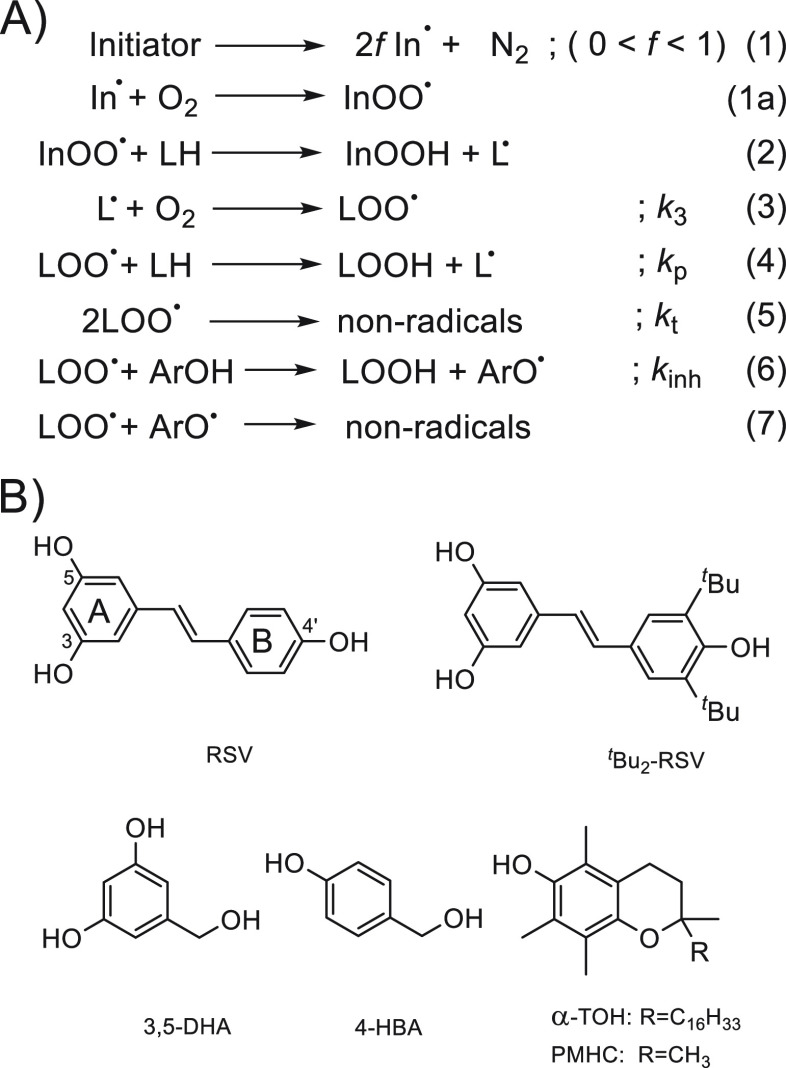
(A) General Mechanism for Autoxidation of Lipids; (B) Structures of Resveratrol (RSV), ^*t*^Bu_2_-RSV, 3,5-Dihydroxybenzyl Alcohol (3,5-DHA), 4-Hydroxybenzyl Alcohol (4-DHA), and 2,2,5,7,8-Pentamethyl-6-hydrochroman (PMHC, an Analogue of α-Tocopherol)

The above mentioned results are rather irrelevant to real peroxidation (Scheme 1A), where alkylperoxyl (lipidperoxyl) radicals LOO^•^ propagate the radical chain of reactions converting lipids (LH) into lipid hydroperoxides, LOOH. The propagation can be stopped by lipid soluble chain-breaking antioxidants (ArOH in reaction 6). In chlorobenzene, RSV reacts with peroxyl radicals rather quickly (*k*_inh_ is 1.4 × 10^5^ and 2.0 × 10^5^ M^–1^ s^–1^) but 16 times slower than α-tocopherol, α-TOH.^16^ Because RSV used together with α-TOH produced an additive induction period, the authors concluded that both antioxidants act separately; i.e., α-TOH is not recycled by RSV in this non-polar, homogeneous model system.^16a^

In dispersed lipid/water systems, RSV is mainly located in the lipid phase or at the lipid/water interface.^17^ The experiments in egg phosphatidylcholine liposomes (egg PC) at pH 6.5 with various initiators (Fe^2+^, lipid soluble, and water-soluble azoinitiators) lead to the conclusion that RSV scavenges LOO^•^ within the liposomal membrane; thus, RSV belongs to the same class of lipophilic antioxidants as α-tocopherol, α-TOH.^18^ RSV inhibits peroxidation of linoleic acid in sodium dodecyl sulfate (SDS) and in cetyltrimethylammonium (CTAB) micelles at pH 7.5, with *k*_inh_ 1.3 × 10^4^ and 0.72 × 10^4^ M^–1^ s^–1^, respectively,^19^ which is 2-fold smaller than *k*_*i*__nh_ obtained for α-TOH in the same systems.

The ability of RSV and its derivatives to trap peroxyl radicals was also studied in unilamellar vesicles (egg PC) by means of a fluorescent probe (BODIPY conjugated with a tocopherol-like sensor),^20^ and the relative rate constants for RSV were at least 2 orders of magnitude smaller than *k*_inh_ for the fluorescent probe. Surprisingly, di-*tert*-butylated derivative (^*t*^Bu_2_-RSV, see Scheme 1B) was a very effective antioxidant reacting 10 and 16 times faster than α-TOH, depending on the initiator. The authors interpreted this striking difference of the reactivity of RSV and its butylated derivative as being due to the better solubility of ^*t*^Bu_2_-RSV in the lipid membrane.

The kinetic parameters presented above confirm the relatively high reactivity of RSV in homogeneous (polar and non-polar) systems, but the results obtained for peroxidation carried out in heterogeneous systems (micelles, liposomes) are confusing and are in contrast to many hypotheses about health benefits assigned to the radical trapping activity of RSV and partially confirmed *in vitro* and *in vivo*. Therefore, we decided to perform a series of peroxidation experiments in micellar and liposomal systems in the pH range 4–10 with RSV used alone and also used together with an equimolar amount of PMHC (Scheme 1B). We also used 3,5-dihydroxybenzyl alcohol (3,5-DHA) and *p*-hydroxybenzyl alcohol (4-HBA) as two compounds with structural motifs resembling rings A and B in RSV; see Scheme 1B. 4-HBA (grastrodigenin) in its glycosylated form known as gastrodin, is able to cross the blood–brain barrier and acts in central nervous system diseases, such as migraine, some cephalalgias, and headache attributed to cranial and cervical vascular disorder. 4-HBA also improves the viability of neural progenitor cells to protect the nervous system against ischemic injury.^21^ 3,5-DHA is a simple analogue of orcinol-type phenols (olivetol, orcinol, cardol).^22^ Additionally, we intended to study the potential synergistic interactions of RSV with PMHC as an analogue of α-TOH, the most active radical trapping natural antioxidant present in living cells.

## Results and Discussion

Comparison of the redox potentials, ionization potentials, and O–H bond dissociation enthalpies of four studied phenols (see Table 1) with parameters for LOO^•^ (*E*° from 1020 to 1110 mV vs NHE at pH 7, and the LOO–H bond dissociation enthalpy (BDE) is 88–90 kcal/mol)^23^ suggests that each of the four phenols will be able to reduce peroxyl radicals. The ionization potential (IP) for each phenol is sufficiently high to avoid a direct electron transfer to molecular oxygen, a process generating O_2_^•–^ when the IP is smaller than 152–154 kcal/mol.^24^ Even the best reducing agent localized outside the biomembranes or micellar lipid phase cannot break the propagation chain, thus, the partition coefficients (log *P*) listed in Table 1 allow us to predict that PMHC and RSV should effectively trap peroxyl radicals within the dispersed lipid phase in contrast to the limited activity of 3,5-DHA and 4-HBA in the lipid phase.

**Table 1:** Literature Values: Redox Potential, *E*° (mV vs NHE), Ionization Potential, IP (kcal·mol^–1^), O–H Bond Dissociation Enthalpy, BDE (kcal·mol^–1^), and Partition Coefficient, log *P*

phenol	*E*°	IP^a^	BDE	log *P*
PMHC	480^25^	154.9^26^	77.3–79.3^27^	3.58^28^
RSV	864/914^b^	161.35^26^	83.7^c^	2.68–3.43^d^
resorcin^e^	810^25^	185.7^29^	88–91^27^	0.76^30^
4-HBA	870^f^	192.05^g^	85.7^27^	0.25^31^

a IP values were calculated by the DFT method in the gas phase.

b Determined in SDS and CTAB micelles at pH 7.4,^19a^ respectively. However, electrochemical studies^32^ in ethanol/water at pH 1–12 indicate the slope −0.45 mV/pH and oxidation potential at pH 7.0 is 634 mV.

c Estimated by the group additivity rule for the weakest O–H.^16b^ The accessible theoretical data are very scattered, from 75.3 to 88.15.^26,33^ We thank the anonymous Reviewer for the critical comment that theoretical values below 80 kcal/mol for RSV are not reliable.

d Values of log *P*: 2.68 (in octanol/water at pH 2),^34^ 3.1 (calculated for pH 6.0),^35^ and 3.43 (determined experimentally)^17^ for partition of RSV between water and LUV formed from 1,2-dimyristoyl-*sn*-glycero-3-phosphocholine (DMPC) at pH 7.4 and 37 °C.

e Data presented for resorcin instead of 3,5-DHA.

f For 4-methylphenol.^36^

g For phenol.^26^

**Figure 1:**
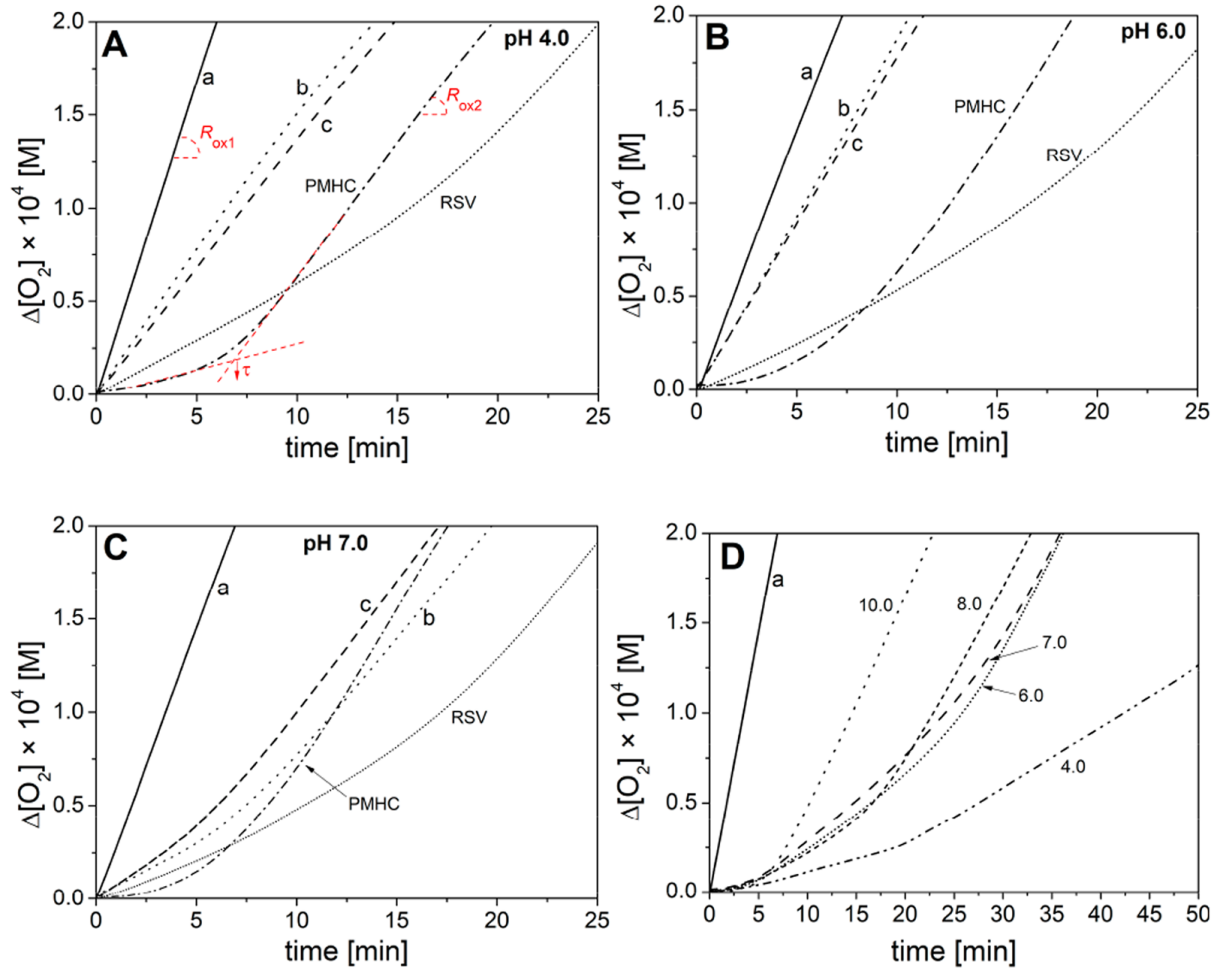
Oxygen uptake for peroxidation of 2.74 mM MeLin in the micelles of 8 mM Triton X-100, initiated with ABAP at 37 °C and pH 4, 6, and 7 (panels A–C). In panel A, the parameters *R*_ox1_, *R*_ox2_, and induction time τ are shown. In each panel, line a denotes spontaneous (non-inhibited) peroxidation and the other lines were recorded for peroxidation in the presence of 1 μM of the following phenols: PMHC, RSV, 3,5-DHA (line b), and 4-HBA (line c). Panel D: equimolar mixture of PMHC/resveratrol at pH 4.0–10.0 (the numbers correspond to pH values). Full size plots for pH 4–10 are presented in the Supporting Information.

Micelles and liposomes are heterogeneous models of biological systems. The first ones are monolayer aggregates of surfactant that enable the studies of reactions proceeding at the water/lipid interface, whereas LUV liposomes structurally resemble bilayers. The general mechanism of peroxidation is presented in Scheme 1A. Water-soluble 2,2′-azobis(2-methylpropionamidine) dihydrochloride (ABAP) was used as a stable, well-defined flux of primary radicals In^•^ (eq 1), immediately reacting with molecular oxygen to give peroxyl radicals (eq 1a) which attack lipid LH (reaction 2) and start the propagation cycle, with very fast addition of O_2_ (reaction 3, *k*_3_ is ∼10^9^ M^–1^ s^–1^) and much slower abstraction of hydrogen atoms (reaction 4, *k*_p_ 0.3–60 M^–1^ s^–1^, depending on the strength of the C–H bond in LH).^24^ Reactions 3 and 4 are repeated tens and hundreds of times; thus, LH are converted into the LOOH until the kinetic chain is terminated (for example, eq 5). The propagation cycle can be stopped by radical trapping agents, as illustrated by reactions 6 and 7 for phenolic antioxidants (ArOH). An effective chain-breaking antioxidant makes a visible suppression of the peroxidation rate called the lag phase or induction phase; see Figure 1A for PMHC. After the antioxidant is depleted, the rate of the process increases; thus, the induction time (τ) can be determined graphically. τ is connected with the rate of initiation (*R*_i_), the starting concentration of the antioxidant [ArOH]_0_, and its stoichiometric factor *n* by eq 8 (eq 8 can be used for determination of any of those parameters if the other three parameters are known).
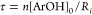
8When τ is determined, the value *k*_inh_ can be calculated from equation:^37^
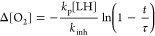
9where Δ[O_2_] is the oxygen consumption measured at time *t* within the induction time, [LH] is the concentration of the lipid subjected peroxidation (here LH = methyl linoleate, MeLin), and *k*_p_ values (reaction 4) were taken: 36 M^–1^ s^–1^ for MeLin in Triton X-100 micelles^38^ and 41 M^–1^ s^–1^ for MeLin in DMPC liposomes^39^). The *R*_i_ values determined from eq 8 for micellar and liposomal systems are collected in the Supporting Information (Table S1).

### Antioxidant Activity in a Micellar System

Typical plots of O_2_ consumption measured during peroxidation of MeLin in the micellar system are presented in Figure 1. The parameters listed in Table 2 indicate that all studied compounds at concentration 1 μM exhibit antioxidant activity at pH 7.0, with an induction period from 6.4 min for 4-HBA to 16.2 min for RSV. Stoichiometric factors (see also Table S2) for RSV at pH 4–8 exceed the value *n* = 1.9 reported by Amorati et al.^16b^ It would be quite reasonable that RSV bearing three phenolic groups is able to trap more than two radicals, as observed by Roginsky for polyphenols with three to eight hydroxyl groups.^40^

**Table 2:** Lengths of Induction Periods, τ, Stoichiometric Factors, *n*, Rate of Inhibited Peroxidation, *R*_inh_, the Slow-Down Factors (*R*_ox_/*R*_inh_, the Ratio of the Rate of the Non-Inhibited Process to the Rate of the Inhibited Process), the Inhibition Rate Constants, *k*_inh_, and Kinetic Chain Lengths, *v*_inh_ = *R*_inh_/*R*_i_, Determined for Autoxidation of 2.74 mM MeLin Dispersed in 8 mM Triton X-100 Micelles in the Presence of 1 μM PMHC, RSV, 3,5-DHA, and 4-HBA or an Equimolar Mixture of PMHC/RSV^a^

pH	τ (min)	*n*^b^	*R*_inh_ (nM s^–1^)	*R*_ox_/*R*_inh_^c^	10^–3^*k*_inh_ (M^–1^ s^–1^)	*v*_inh_
PMHC						
4.0	7.2 ± 0.1	2.0	35 ± 4	15.8	10.9 ± 2.2	7.6
6.0	7.2 ± 0.6	2.0	52 ± 7	9.1	6.7 ± 1.3	11.1
7.0	7.6 ± 0.7	2.0	37 ± 5	9.6	18.8 ± 3.8	8.4
8.0	7.5 ± 0.5	2.0	47 ± 5	7.5	17.0 ± 3.4	10.4
10.0	5.8 ± 0.7	2.0	37 ± 5	7.5	29.2 ± 5.8	6.5
RSV						
4.0	15.6 ± 0.8	4.3	94 ± 6	5.9	1.5 ± 0.3	20.4
6.0	17.2 ± 0.7	4.9	103 ± 12	4.6	1.2 ± 0.2	21.9
7.0	16.2 ± 1.1	4.3	90 ± 6	4.0	1.5 ± 0.3	20.5
8.0	11.4 ± 0.2	3.1	81 ± 6	4.3	2.5 ± 0.5	18.0
10.0	4.6 ± 0.4	1.6	124 ± 13	2.2	3.7 ± 0.7	21.8
3,5-DHA						
4.0			231 ± 1^d^	2.4^d^	e	50.2
7.0	6.8 ± 0.3	1.8	109 ± 4	3.3	3.0 ± 0.6	24.8
4-HBA						
4.0			215 ± 3^d^	2.6^d^	e	46.7
7.0	6.4 ± 0.2	1.7	117 ± 5	3.1	2.8 ± 0.6	26.6
PMHC/RSV						
4.0	21.5 ± 0.5	3.9^f^	22 ± 5	25.2	g	4.8
6.0	25.7 ± 1.3	5.2	67 ± 6	7.0		14.3
7.0	23.4 ± 1.7	4.2	66 ± 8	5.4		15.0
8.0	18.5 ± 0.8	3.0	66 ± 14	5.3		14.7
10.0	6.6 ± 0.7	0.3	21 ± 4	13.1		3.7

a The experiments were performed at 37 °C and pH 4.0, 6.0, 7.0, 8.0, and 10.0. Peroxidation was initiated by 10 mM ABAP. Values are expressed as the mean ± standard deviation (SD).

b For α-TOH and PMHC, *n* = 2.0.

c The *R*_ox_/*R*_inh_ ratio informs how many times the inhibited oxidation is slower than the spontaneous (non-inhibited) process (for *R*_ox_ as well as *R*_i_ values, see Table S1).

d There was no inhibition period for 3,5-DHA and 4-HBA at pH 4, 5, and 6. *R*_inh_ means rate of retardation; see Figure 1A.

e For these systems, the inhibition time was not detected and *k*_inh_ could not be calculated.

f For PMHC/RSV, the parameter *n* corresponds to RSV and was calculated from the equation *n*_RSV_ = *R*_i_(τ_PMHC/RSV_ – τ_PMHC_)/[RSV] adapted from ref (41).

g *k*_inh_ cannot be calculated for mixed antioxidants.

3,5-DHA and 4-HBA were not active at pH 4–6; thus, *n* ∼ 4 for RSV at pH 4–6 cannot be explained as a sum of *n* for 3,5-DHA and 4-HBA. Later in this manuscript, we will provide another explanation for high *n* for RSV. At pH 7 (Figure 1C), the induction time was observed for both 3,5-DHA and 4-HBA and the calculated values *n* are close to 2 (Table 2). However, neither 3,5-DHA nor 4-HBA are active inhibitors because they moderately suppress the rate of oxidation (the *R*_ox_/*R*_inh_ ratio and ν_inh_ are relatively high).

PMHC and RSV do not change their reactivities (*k*_inh_, *n*, *R*_inh_) at pH 4–7 (Table 2). The only exception is that τ generated by an equimolar mixture of RSV/PMHC (25.7 min at pH 6.0) is 12% longer than the sum of individual induction periods.^42^ When passing to alkaline conditions (pH 8 and 10), τ and *n* parameters decreased while *k*_inh_ increased (see also Figure S1 with the oxygen uptake at pH 8 and 10, not shown in the main manuscript). A similar but less pronounced effect was observed for PMHC, and the synergy of PMHC/RSV was lost; see Figure 1D.

### Antioxidant Activity in Liposomes

**Figure 2:**
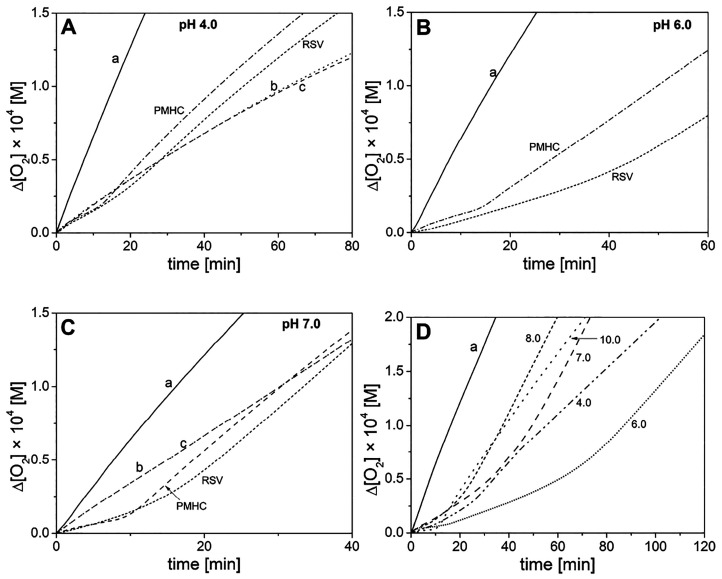
Oxygen uptake during peroxidation of 2.74 mM MeLin in liposomes (20.2 mM DMPC) initiated with ABAP, at 37 °C. Panels A–C: peroxidation at pH 4, 6, and 7. Line assignments: non-inhibited peroxidation (line a), 3,5-DHA (line b), 4-HBA (line c), RSV, and PMHC are marked directly on the plots. Panel D: effect of an equimolar mixture (1 μM each) of PMHC/resveratrol for the processes carried out at pH 4.0–10.0 (the numbers correspond to pH values). Full size plots for pH 4–10 are presented in the Supporting Information.

Plots of O_2_ uptake recorded during autoxidation on MeLin in DMPC liposomes are shown in Figure 2 (and Figure S2 in the Supporting Information). The rates of initiation are in the range 2.3–3.8 nM s^–1^ (see Table S1); thus, pH does not affect *R*_i_. However, it is worth mentioning that, regardless of a comparable *R*_i_ in both micellar and liposomal systems, peroxidation of MeLin carried out in liposomes is 2-fold slower than in the micellar system. We discussed this problem and possible explanations in our recent paper.^43^

The kinetic profiles of oxygen uptake in the presence of 1 μM of studied phenols or an equimolar mixture of PMHC/RSV are included in Figure 2 (for alkaline pH, see the Supporting Information), and the kinetic parameters are collected in Table 3. The inhibition rate constant 14,000 ± 2700 M^–1^ s^–1^ for PMHC (Table 3) is in good agreement with *k*_inh_ determined for PMHC in DLPC liposomes, 17,800 ± 1400 M^–1^ s^–1^,^37^ as well as with our previous works.^43,44^ 3,5-DHA and 4-HBA are not effective antioxidants in the liposomal system at concentration 1 μM because there is no evident inhibition period, and both compounds cause a retardation at pH 4 and 7.

**Table 3:** Lengths of Induction Periods, τ, Stoichiometric Factors, *n*, Rate of Inhibited Peroxidation, *R*_inh_, the Slow-Down Factors (*R*_ox_/*R*_inh_, the Ratio of the Rate of the Non-Inhibited Process to the Rate of the Inhibited Process), the Inhibition Rate Constants, *k*_inh_, and Kinetic Chain Lengths, *v*_inh_ = *R*_inh_/*R*_i_, Determined for Autoxidation of 2.74 mM MeLin in 20.2 mM DMPC Liposomes in the Presence of 1 μM PMHC, RSV, 3,5-DHA, and 4-HBA or an Equimolar Mixture of PMHC/RSV^a^

pH	τ (min)	*n*^b^	*R*_inh_ (nM s^–1^)	*R*_ox_/*R*_inh_^c^	10^–3^ *k*_inh_ (M^–1^ s^–1^)	*v*_inh_
PMHC						
4.0	10.9 ± 0.6	2.0	18 ± 3	4.7	12.8 ± 2.5	5.8
6.0	14.6 ± 0.5	2.0	19 ± 3	4.6	7.9 ± 1.6	8.3
7.0	8.6 ± 0.7	2.0	20 ± 4	5.0	14.0 ± 2.7	5.3
8.0	9.8 ± 0.5	2.0	22 ± 5	4.5	12.3 ± 2.5	6.5
10.0	9.4 ± 0.7	2.0	16 ± 3	4.3	16.1 ± 3.3	4.4
RSV						
4.0	18.1 ± 0.9	3.4	24 ± 5	3.5	4.9 ± 1.1^d^	7.7
6.0	40.8 ± 1.0	5.6	18 ± 3	4.8	3.3 ± 0.5^d^	7.8
7.0	14.8 ± 0.6	3.4	22 ± 2	4.5	7.1 ± 1.4^d^	5.8
8.0	13.6 ± 2.0	2.8	35 ± 5	2.9	5.5 ± 1.1^d^	10.3
10.0	-	-	48 ± 5^e^	1.4^e^	-f	13.3
3,5-DHA						
4.0	-	-	33 ± 5^e^	2.5^e^	-f	10.6
7.0	-	-	73 ± 3^e^	1.4^e^	-f	19.2
4-HBA						
4.0	-	-	33 ± 6^e^	2.5^e^	-f	10.6
7.0	-	-	64 ± 9^e^	1.5^e^	-f	16.8
PMHC/RSV						
4.0	33.1 ± 4.0	4.1^g^	17 ± 4	4.9	-h	5.5
6.0	64.4 ± 2.1	6.9	14 ± 3	6.2	-	6.1
7.0	40.8 ± 1.4	7.3	18 ± 5	5.5	-	4.7
8.0	21.3 ± 2.8	2.3	16 ± 4	6.3	-	4.7
10.0	10.8 ± 0.3	0.3	12 ± 3	5.8	-	3.3

a The experiments were performed at 37 °C and pH 4.0, 6.0, 7.0, 8.0, and 10.0. Peroxidation was initiated by 10 mM ABAP. Values are expressed as the mean ± standard deviation (SD).

b For α-TOH and PMHC, *n* = 2.0.

c The *R*_ox_/*R*_inh_ ratio informs how many times the inhibited oxidation is slower than the spontaneous (non-inhibited) process (for *R*_ox_ as well as *R*_i_ values, see Supporting Information).

d One of the Reviewers pointed out that *k*_inh_ calculated from eq 9 represents the rate constant with the assumption that *n* = 2.0; therefore, eq 9 used for antioxidants with higher capacity (*n* > 2) gives the minimal, apparent *k*_inh_, but the real value might be obtained by multiplication of *k*_inh_ by factor “*n*/2”. The values presented in this table for RSV have not been corrected and represent the minimal *k*_inh_.

e There was no inhibition period. *R*_inh_ means rate of retardation; see Figure 2A,C.

f For these systems, the inhibition time was not detected and *k*_inh_ could not be calculated from eq 9.

g For PMHC/RSV, parameter *n* corresponds to RSV and was calculated from the equation *n*_RSV_ = *R*_i_(τ_PMHC/RSV_ – τ_PMHC_)/[RSV] adapted from ref (41).

h *k*_inh_ cannot be calculated for mixed antioxidants.

The ability of RSV to inhibit peroxidation of MeLin/DMPC liposomes was measured in the extended range of pH. There is no induction time at pH 10.0, and the *R*_ox_/*R*_inh_ ratio (expressing how many times the inhibited oxidation is slower than spontaneous, non-inhibited peroxidation) is 1.4, indicating a retardation of autoxidation. A hypothetical explanation of a shorter induction period at pH 10 can be a fast decomposition of RSV in the alkaline system because the stability of RSV is strongly pH dependent. Its half-life ranges from almost a year at pH 1.2 to 20 days at pH 6.8, but it exponentially decreases at basic pH’s: 2 days at pH 7.4, hours at pH 8, and minutes at pH 9 and 10.^45^

In contrast to the results obtained by Pratt et al.^20^ (see the Introduction),^46^ we observed a clear inhibition effect of RSV at pH 7.0, as well as in the whole pH range 4–8. Surprisingly, at pH 6.0, the extremely long τ = 40.8 min was recorded, being 2 or 3 times longer than at pH 4, 7, and 8 (Figure 2B vs Figure 2A and C). Several repetitions confirmed that RSV is more efficient (see the τ, *n*, and *R*_inh_ parameters in Table 3) at this particular pH. In our previous work, we documented that the Clark type electrode works correctly at pH 4–10 in dispersed lipid/water systems,^43,47^ with the only deviations reported at extremal pH, but not in the pH range 5–8, which is of particular interest to biochemists and food chemists. Here, regardless of the value of pH, the parameter *R*_i_ is still within the range 4.4–5.7 nM s^–1^ in micelles and 2.3–3.8 nM s^–1^ in liposomes (see Table S1). Therefore, the observed effect at pH 6 and 7 is not due to a specific (slower) rate of initiation.

We checked the hypothesis that such a peculiar effect might correlate with ionization of RSV and its pH dependent localization in the DMPC membrane. Ionization of a phenolic antioxidant facilitates the fast electron transfer instead of the much slower HAT process and is well documented for electron deficient radicals in ionization supporting solvents.^48^ Another possibility is that deprotonation of OH might cause a decrease of the BDE of the remaining hydroxyl in polyphenols.

There is a general agreement that the 4′-OH group is the most acidic site in RSV (supported by NMR titration^34^). Table S5 in the Supporting Information collects 12 accessible values of p*K*_a1_ for RSV, ranging from 6.4 to 9.7. Although p*K*_a1_ ∼ 6 would be a tempting explanation for the peculiar antioxidant effect of RSV at pH 6 in liposomes, we have to exclude such a low p*K*_a1_ because none of the structural fragments in RSV is able to increase the acidity of RSV to be comparable to (or stronger than) the acidity of phenols with strongly electron withdrawing groups: 4-NO_2_-phenol (7.15), 4-CN-phenol (7.97), 4-OH-benzaldehyde (7.6), or 4-OH-acetophenone (8.05).^49,50^ We disregarded the unusually low p*K*_a_ as erroneously determined for the first excited singlet state RSV* and contaminated by the products of possible photoizomerization^51^ (see the Supporting Information), and we believe that p*K*_a_ ∼ 9.0 (determined by NMR titration and close to the theoretical one^10e^) is the most relevant to the acidity of RSV in water. Such a relatively high p*K*_a_ perfectly supports the localization of a large fraction of RSV in the lipid bilayer at pH ≤ 8, the partition coefficient determined by Neves et al.^17^ in LUV/DPMC (log *D* = 3.43, see Table 1), and should not be dramatically changed at lower pH.

**Scheme 2:**
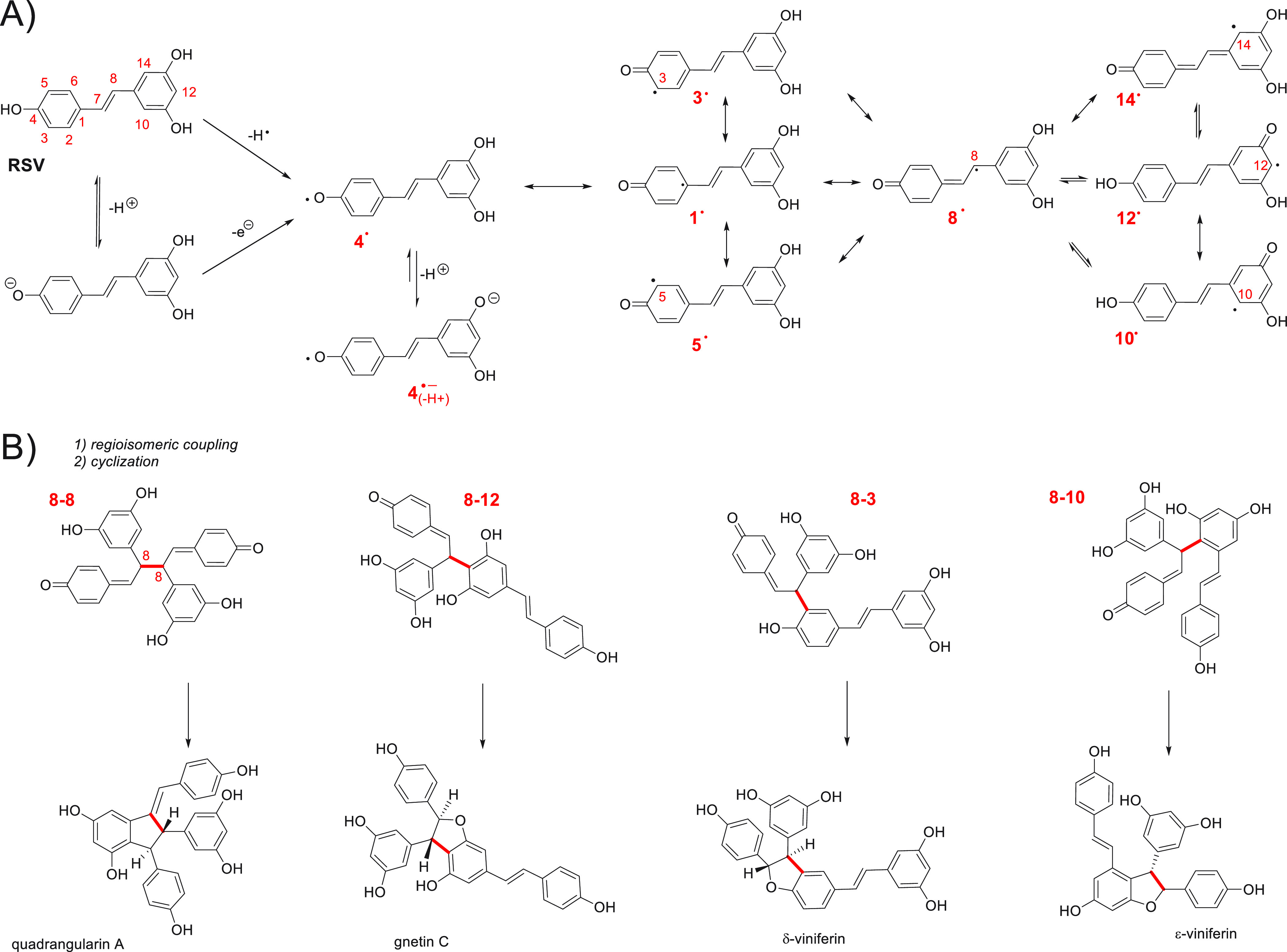
(A) Formation of RSV Phenoxyl Radical (**4^•^**) Proceeding via Direct HAT (Preferred Route) and a SPLET-Like Mechanism (Not Preferred at pH < 7 due to the Relatively High p*K*_a_; see the Results and Discussion); (B) Products of Regio- and Stereospecific Coupling of **8**^•^ with **3^•^**, **10^•^**, and **12^•^** with Subsequent Cyclization of the Formed Dimers^55^

Because the problem of the best antioxidant activity of RSV at pH 6 cannot be explained by the dramatic change of localization/ionization of RSV at pH 6 ± 1, another explanation is probable—the formation of aggregates of RSV that will facilitate the reaction of coupling of phenoxyl radicals and the formation of dimeric products with the recovered ability to trap radicals.^52^ RSV (in solutions) forms aggregates, and the minimal concentration of RSV is pH dependent (12.5 μM at pH 5.5 and 37 μM at pH 10.5).^53^ Although the reliability of such spectrofluorimetric results was questioned,^51c^ the presence of aggregates was confirmed with the MALDI technique^51b^ but at pH > 7.^54^ In dispersed systems, RSV acts at the interface of lipid/water;^17^ therefore, an increased local concentration of RSV molecules and their initial reactions with peroxyl radicals might trigger a cascade of processes illustrated in Scheme 2.

In plants, RSV is biotransformed to dimers and then to higher oligomers to rapidly generate novel defense compounds in response to pathogenesis. Possible pathways of such biosynthesis as well as laboratory methods for synthesis are described (among many others) in a comprehensive review by Stephenson and co-workers.^55^ The resveratrol dimers are almost universally generated by an oxidative radical coupling as a part of biosynthesis in plant, whereas in a laboratory a variety of oxidation strategies are used including enzymatic, organic, inorganic, and photochemical oxidations. Formation of δ-viniferin as a product of cyclization of 8–3 dimer produced from RSV was reported upon the reaction with dpph^•^ radicals in methanol (18% yield)^9a^ and the reaction with galvinoxyl radicals in ethanol, with 41% yield^12^ (the author did not analyze other products of dimerization). Moreover, electrochemical measurements of the pH dependence of RSV oxidation indicated a non-Nernstian slope, interpreted as being due to possible dimerization (see footnote *b* in Table 1).^32^ Stephenson et al. described also the examples of the pH-sensitive nature of the dimerization of resveratrol and its impact on product distribution. For example, oxidation catalyzed by horseradish peroxidase in 1:1 acetone/water gives at pH 8 selectively formed *trans*-δ-viniferin (93%), but a dramatic change in product distribution is observed at pH 6.0 (1:1 mixture of leachinols F/G), at pH 5 pallidol (a product of double cyclization of 8–8 dimer, alternative to quandrangularin A), and at pH 4 *cis*-δ-viniferin.^56^

Serendipidously, our experimental results also indicate that pH 6 appears as a boundary region at which RSV exhibits some exceptional behavior, and we suggest that this effect is due to the high acidity of resveratryl radical **4^•^** undergoing fast deprotonation to a much more polar radical anion (see Scheme 2A) that might affect the localization and reactivity of the radicals in the lipid phase. An example of such an increase of acidity can be found for HO–C_6_H_4_–C_6_H_4_–O^•^ radical (p*K*_a_ = 7.5 ± 0.1) and its parent 4,4′-bisphenol with p*K*_a_ = 9.5.^57,58^ We estimate that the p*K*_a_ for ****4^•^**** should not be higher than 7.0 (because RSV is a bit stronger acid than 4,4′-bisphenol) and not lower than 6.5, because spectra of **4^•^** measured at the pH range from 1.0 to 6.5 are identical and have the same decay profile,^59^ indicating a neutral form of **4^•^** at pH < 6.5. In the same work, Kerzig et al.^59b^ reported the half-life of ****4^•^**** as 50 μs (in water). Therefore, at pH ∼ 6–7, two forms of radicals, **4^•^** and **4^•^**^–^, are present in the system, facilitating the formation of dimers.

The four dimers presented in Scheme 2B are examples of radical–radical coupling (with participation of **8^•^** as one of the components), but the possible number of combinations and the products of cyclization are very high.^60^ Remaining with these four particular examples of dimers: three of them recovered their OH groups at the (former) 4-position that are prone to exhibit the same (dimers 8–10 and 8–12) or better (dimer 8–3, with an *ortho*-alkyl substituent) radical trapping activity than 4′-OH in RSV. Thus, the products of oxidation might still be effective chain-breaking antioxidants (!), resulting in longer induction time (and the stoichiometric coefficient *n* calculated from τ, as can be observed in Tables 2 and 3).

**Scheme 3:**
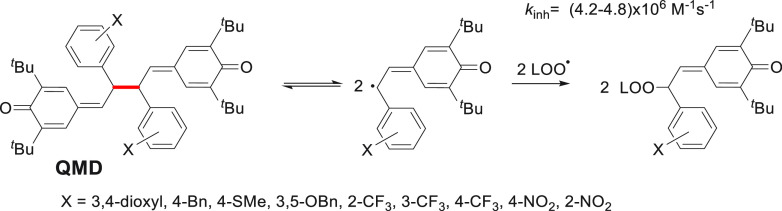
Dissociation of Series of Quinone Methide Dimers (QMDs) and Their Fast Reaction with Peroxyl Radicals^61b^

One of the presented dimers, 8–8, is exceptionally interesting because, unlike other dimers, the groups 4-OH are not recovered during dimerization. However, this quinone methide 8–8 dimer might be more active than the parent RSV, as has been reported by Pratt, Stephenson, and co-workers for the *tert*-butylated analogue, QMD; see Scheme 3.^20,61^

QMD reacts with peroxyl radicals faster than α-TOH regardless of the electron withdrawing or donating character of X groups; see *k*_inh_ in Scheme 3. Pratt et al. tried to explain the high antioxidant activity of QMD in non-polar solvents, and they excluded several mechanisms like HAT from the C–H bond, tautomerization, and direct addition of LOO^•^ to QMD. Instead, the authors documented homolysis of the weak central C–C bond (shown in Scheme 3 in red) followed by combination of the resultant persistent phenoxyl radicals with peroxyl radicals.^61b^ Unfortunately, the high antioxidant activity observed for QMD in solvents is lost during peroxidation carried out in egg PC liposomes, and the authors explained such a disappointing effect as being because of localization in depth, with no contact with polar heads of PC.^61b^ We suppose that dimer 8–8 formed from RSV is less polar and its four resorcinoid hydroxyl groups can be H bonded to polar heads that would make additional strain and facilitate the dissociation of the C–C bond in 8–8.

### Synergism of RSV and PMHC

**Figure 3:**
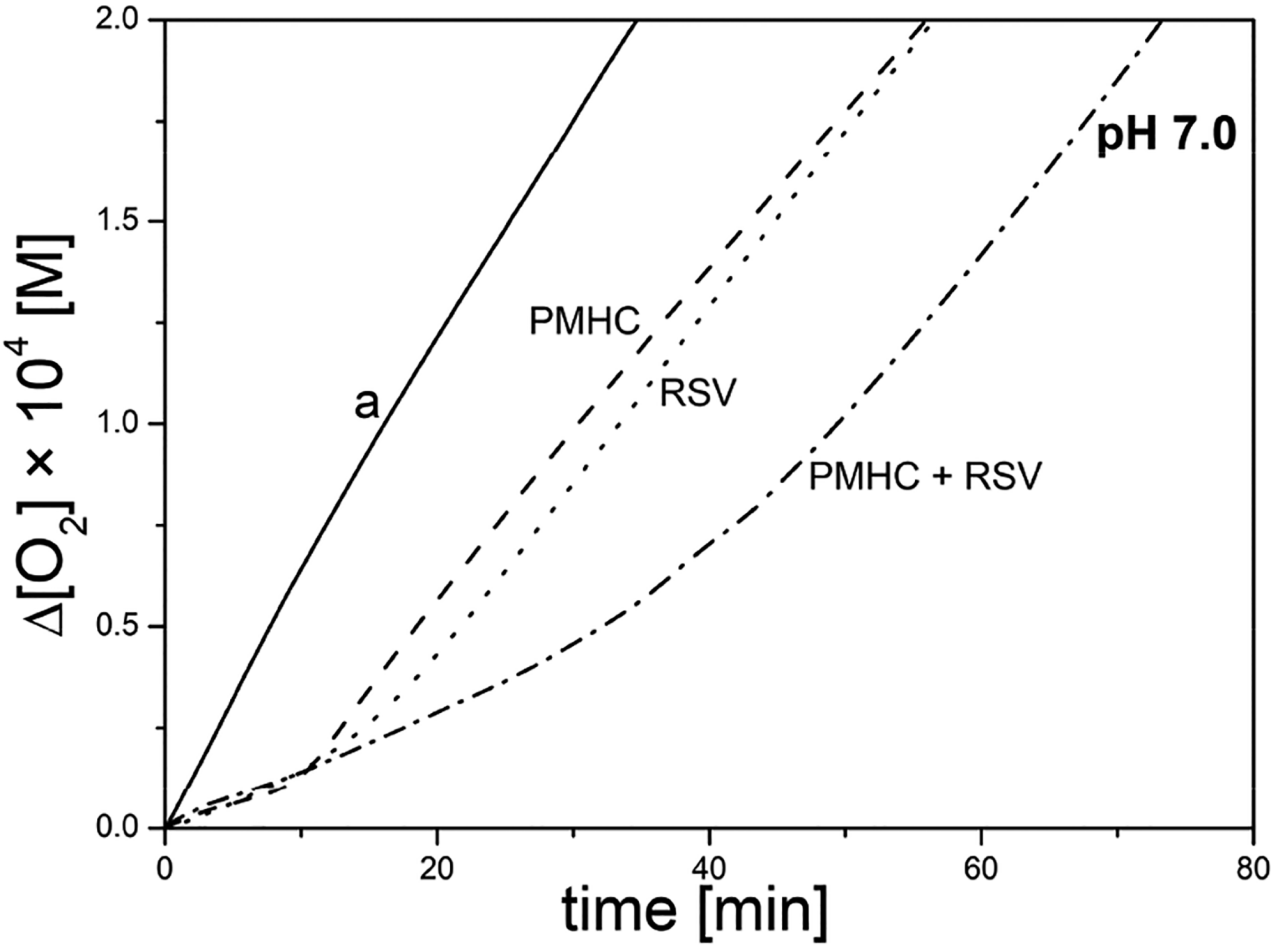
Oxygen uptake for peroxidation of MeLin/DMPC at pH 7.0: without inhibitor (curve a), with 1 μM PMHC and RSV and a mixture of 1 μM PMHC/1 μM RSV. The experimental conditions are the same as those in Figure 2.

Cooperation of α-tocopherol and RSV was the subject of some opposed reports. In 2002, Fang and co-workers^19a^ demonstrated that peroxidation of linoleic acid in micellar systems (SDS and CTAB) was effectively inhibited by RSV used alone but also in equimolar combination with α-TOH. A year later, Amorati^16a^ and co-workers pointed out that the reported “superb” antioxidant activity of RSV is too high as for a phenol with BDE = 83.7 kcal/mol for 4′-OH (see Table 1) being about 3.1 kcal/mol higher than the BDE for α-TOH. In the same work, the authors reported that RSV and 2,6-di-*tert*-4-methylphenol (BHT) were not able to recycle α-TOH, in contrast to 2,6-di-*tert*-4-methoxyphenol (BHA) which regenerated α-TOH (reaction 10) with the rate constant *k*_r_ = 5.9 × 10^3^ M^–1^ s^–1^ (which is slightly above *k*_r_ ∼ 10^3^ M^–1^ s^–1^ proposed as the minimum for sufficient regeneration).^41,62^
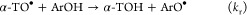
10The conclusion that resveratrol in homogeneous solution is neither an outstanding antioxidant nor co-antioxidant was confronted with results in heterogeneous systems reported by Fang and Zhou,^19b^ who demonstrated the *additive inhibition* in SDS micelles (τ_RSV+PMHC_ = τ_RSV_ + τ_PMHC_) and *hyper-additive inhibition* (synergy: τ_RSV+PMHC_ > τ_RSV_ + τ_PMHC_) in CTAB micelles. They were looking for an explanation that would not be in contradiction to homogeneous systems. With the assumption that RSV is deprotonated at pH 7.4 (supported by erroneous p*K*_a_ = 6.4) and looking at the relatively low oxidation potentials (see Table 1), Fang and Zhou proposed a complementary mechanism of regeneration of α-TOH including the electron transfer from RSV anion to α-TO^•^, with a subsequent protonation, α-TO^–^ + H^+^ → α-TOH. Because the real p*K*_a_ is higher (see the Supporting Information), we decided to examine the synergistic effects for RSV/PMHC in both micellar and liposomal systems, in an extended range of pH; see Figures 1D and 2D and Tables 2 and 3. The induction period 23.4 min recorded at pH 7 in Triton X-100 is an *additive inhibition* and is in good agreement with that reported by Fang et al. in SDS micelles at pH 7.4.^19a^ In a liposomal system, the induction periods τ determined at pH 4 and 8 are (within the experimental errors) almost the sum of individual τ’s for RSV and PMHC used separately. To our surprise, at pH 6 and 7, the hyper-additive inhibition is observed, and at pH 6, the system is protected for 64.4 min (20% prolongation versus 55.4 min expected as the sum of individual contributions). At pH 7, the astonishing hyper-inhibition with τ_RSV+PMHC_ = 40.8 ± 1.4 min is observed, being 2 times longer than the sum of individual contributions of RSV and PMHC when used separately; see Figure 3.

The evident prolongation of induction period τ without any substantial decrease of the rate of inhibited peroxidation (*R*_inh_) compared to *R*_inh_ for the systems inhibited by PMHC used alone is an argument that PMHC is a leading antioxidant (responsible for breaking the chain and suppressing the rate to *R*_inh_), whereas RSV is a co-antioxidant. Indeed, the rate constants *k*_inh_ presented in Tables 2 and 3 suggest that PMHC in the micellar system is about 10 times more reactive toward lipidperoxyl radicals than RSV, whereas in phospholipid LUVs the difference in reactivity decreases to a factor of 2 or 3, but PMHC is still more reactive than RSV. We have to exclude the explanation suggested by Fang and Zhou^19b^ that at pH > 6 a large fraction of RSV is ionized and prone to reduce the α-TO^•^ radical (or its PMHC analogue) because the recommended p*K*_a_ is 9.0 (see Table S5 and the discussion about p*K*_a_). If an anion would play any significant role in regeneration of PMHC, the synergy would be better at pH > 8 than at pH 4–7, but such an improvement is not observed (even if RSV undergoes faster decomposition at pH 9 and 10, some fraction of anions would be able to regenerate PMHC). We have three arguments for reasoning the observed synergy. At first, the difference between the O–H BDE for RSV and α-TOC might be smaller than 5 kcal/mol (see footnote *c* in Table 1). Second, the endoergic character of the reaction does not exclude the process. For example, H atom abstraction, *t*-BuOO^•^ + PhOH → *t*-BuOOH + PhO^•^, is described by Δ*G*° = +4 kcal/mol and *k* = 2.8 × 10^3^ M^–1^ s^–1^ (BDE_ROO–H_ is ∼88 kcal/mol).^24^ Also, the reactions of dpph^•^ (BDE_N–H_ = 78.9 kcal/mol) with the majority of phenols (BDE_O–H_ within the range 78–88 kcal/mol) are endoergic, but the reaction (reversible) is shifted to the products because ArO^•^ radicals are consumed in other processes.^63^ A similar mechanism might operate for the reaction of RSV with α-TO^•^. A third argument is that the half-life of the α-TO^•^ radical is greatly prolonged when passing from homogeneous non-polar systems to lipid/water dispersions: the rate constant for self-decay of α-TO^•^ in benzene, expressed as 2*k*_TO^•^_, is 5.9 × 10^3^ M^–1^ s^–1^,^64,65^ whereas in CTAB and SDS micelles 2*k*_TO^•^_ is 3 orders of magnitude smaller (15 and 75 M^–1^ s^–1^, respectively).^19b^ For such long living radicals, the effective regeneration should be possible, and values of *k*_r_ = 20 M^–1^ s^–1^ in CTAB and 19 M^–1^ s^–1^ in SDS measured by Fang and Zhou^19b^ seem to be sufficient for effective regeneration of α-TOH in CTAB (*k*_r_ ∼ 2*k*_TO^•^_) and in SDS (*k*_r_ ∼ 25% of 2*k*_TO^•^_). Our experimental results indicate closer physical and kinetic similarity of DMPC liposomes to CTAB (the same surface charge, the synergy detected in both systems) than to SDS micelles, and we assume that the rate constants for self-decay of tocopheroxyl radical in DMPC liposomes and in CTAB micelles will be the same or similar. Moreover, regeneration is facilitated due to the proximity of molecules of antioxidant and co-oxidant within the same lipid phase (vide supra and see also log *P* parameters in Table 1). The observed contradiction between the results obtained in homogeneous non-polar systems and in heterogeneous water/lipid/surfactant systems is an additional example of the high impact of microenvironment on the overall activity and cooperativity of antioxidants and co-antioxidants.

## Conclusions

We observed pH dependent effectiveness of RSV as an inhibitor of peroxidation of methyl linoleate in Triton X-100 micelles and in DMPC liposomes (LUVs). The best activity of RSV was at pH 6, with an outstanding increase of effectiveness in liposomes. The observed effects cannot be explained by a direct reaction of deprotonated RSV with lipidperoxyl radicals. Based on recent progress in the chemistry of natural and synthetic derivatives of RSV and its dimers,^20,61^ we propose the plausible mechanism in which peroxyl radicals abstract H atoms from RSV molecules to form persistent radicals which subsequently form dimers (and further cyclization products), with recovered hydroxyl groups able to trap peroxyl radicals. The formation of such dimers is facilitated in micellar and liposomal systems due to the increased concentration of RSV at the lipid–water interface at pH 6–7.

We also rationalized the inconsistency on cooperative interaction of RSV with α-tocopherol in micellar systems^19b^ versus the lack of synergy in non-polar solvents.^16a^ The increased persistency of tocopheroxyl radical in dispersed lipid/water systems and localization of RSV within the lipid region facilitate the probability of reaction of RSV with tocopheroxyl radical, resulting in recovery of α-TOH. The presented results indicate a great importance of solvent and microenvironment effects for the overall kinetics of radical reactions as well for interpretation of mechanisms of radical trapping by natural compounds.

## Experimental Section

### Chemicals

Chemicals and solvents for kinetic measurements were purchased from Sigma-Aldrich, TCI, and Avanti Polar Lipids Inc. and were used without any additional purification: 2,2′-azobis(2-amidinopropane) dihydrochloride (ABAP; 97%, Sigma-Aldrich), Triton X-100 (polyethylene glycol *p*-(1,1,3,3-tetramethylbutyl)-phenyl ether; 98%, Sigma-Aldrich), methyl linoleate (MeLin; 99%, Sigma-Aldrich), 1,2-dimyristoyl-*sn*-glycero-3-phosphocholine (DMPC; 99%, Avanti Polar Lipids Inc.), 2,2,5,7,8-pentamethylchroman-6-ol (PMHC; 99%, Sigma-Aldrich), 3,4′,5-trihydroxy-*trans*-stilbene (resveratrol; >99%, TCI), 3,5-dihydroxybenzyl alcohol (99%, Sigma-Aldrich), and 4-hydroxybenzyl alcohol (99%, Sigma-Aldrich).

### Preparation of Micelles

The micelles were prepared following the method described in our previous kinetic studies.^43,47a^ Glass test tubes with 10 μL of methyl linoleate (MeLin) and 5.5 mL of 16 mM Triton X-100 were stirred on vortex for 60 s. Next, 5.5 mL of buffer solution (pH 4.0, 6.0, 7.0, 8.0, or 10.0) was added, and the mixture was stirred again for 60 s. Apart from pH 4, all buffers were prepared from inorganic components: pH 4 (acetic), pH 6.0, 7,0, and 8.0 (phosphate), and pH 10.0 (borate). The final concentration of MeLin and Triton X-100 in the micellar system was 2.74 mM for lipid and 8 mM for surfactant.

### Preparation of Liposomes—Large Unilamellar Vesicles (LUVs)

LUVs were obtained from multilamellar vesicles (MLVs) by a previously described extrusion procedure performed in a small volume extrusion apparatus described in our previous kinetic studies.^44^ A 65.3 mg portion of 1,2-dimyristoyl-*sn*-glycero-3-phosphocholine (DMPC) was dissolved in 1.5 mL of CHCl_3_ in a round-bottom flask. Then, 4 μL of MeLin was added, the solvent was removed using a rotary evaporator, and the lipids were vacuum-dried overnight. Next, the obtained lipid film was suspended in buffers of pH 4.0, 6.0, 7.0, 8.0, and 10.0; the final concentration of lipids was 2.74 mM MeLin and 20.2 mM DMPC. LUVs were obtained by multiple extrusions (at least 21 times) in an Avanti mini extruder (Avanti Polar Lipids Inc.). Basing on the DLS method, the size distribution of LUVs was determined, and it was 170 ± 45 nm (in agreement with previously prepared LUVs^43^). The buffer solutions were the same as those for micellar systems.

### Methodology of Autoxidation Measurements

The rate of peroxidation of MeLin dispersed in micellar and liposomal model systems was monitored as the oxygen uptake using a Biological Oxygen Monitor (Yellow Springs Instruments equipped with a Clark-type electrode by the methodology described in our previous papers).^44,47a^ The chambers with magnetic stirring containing 5 mL of micelles or 2 mL of liposomes were saturated with oxygen; then, the electrodes were placed inside the chambers and peroxidation was initiated by injection of an aqueous solution of ABAP (final concentration 10 mM). After 10% of oxygen was consumed, 10 μL of methanolic solution of PMHC, resveratrol, 3,5-dihydroxybenzyl alcohol, 4-hydroxybenzyl alcohol, or an equimolar mixture of PMHC/resveratrol was added (the final concentration of each tested compounds was 1 μM).

## Supporting Information Available

Kinetic traces for oxygen uptake during peroxidation of methyl linoleate in micellar and liposomal systems at pH 4, 6, 7, 8, and 10 inhibited by 1 μM PMHC, RSV, 3,5-DHA, 4-HBA, and equimolar RSV/PMHC (Figure S1A-F, Figure S2), the rates of initiation, *R*_i_, the rates of the non-inhibited process, *R*_ox1_, kinetic chain lengths, ν_ox1_, determined for peroxidation of methyl linoleate in micelles and in liposomes (Table S1), the stoichiometric factors and parameters used for their calculation (rates of initiation, *R*_i_, lengths of induction period, τ) in micellar (Table S2) and in liposomal systems (Table S3), data for PMHC/3,5-DHA (Figure S4, Table S4), p*K*_a_ values for RSV (Table S5) and discussion about the acidity of RSV (PDF)
