# Supplementary material for: Unexpected Role of pH and Microenvironment on the Antioxidant and Synergistic Activity of Resveratrol in Model Micellar and Liposomal Systems
Source: J Org Chem. 2021 Nov 29;87(3):1698–709. doi: 10.1021/acs.joc.1c01801 (PMC8822491; doi:10.1021/acs.joc.1c01801)
Supplement: Supplementary file 1 — jo1c01801_si_001.pdf [file jo1c01801_si_001.pdf]

SUPPORTING INFORMATION

Unexpected role of pH and microenvironment on the antioxidant and synergistic activity of resveratrol in model micellar and liposomal systems.

Adrian Konopko[1,2] & Grzegorz Litwinienko[1,*]

[1] University of Warsaw, Faculty of Chemistry, Pasteura 1, 02-093 Warsaw, Poland.

[2] Nencki Institute of Experimental Biology, Polish Academy of Sciences, 3 Pasteur St., Warsaw, 02-093, Poland

* E-mail: litwin@chem.uw.edu.pl

	description	page
**Figure S1.**	Plots for peroxidation of MeLin[a] in Triton X-100 at pH 4, 6, 7, 8, 10.	**S-2**
**Figure S2.**	Plots for peroxidation of MeLin[a] in DMPC[a] liposomes at pH 4, 6, 7, 8, 10.	**S-3**
**Figure S3.**	Plots for peroxidation of MeLin[a] in DMPC liposomes at pH 6.0 and 7.0: without inhibitor (curve a), with 1 μM of: PMHC[a], RSV and equimolar mixture of PMHC/RSV.	**S-4**
**Figure S4.**	Plots for peroxidation of MeLin[a] in Triton X-100 at pH 4.0-7.0:, without inhibitor, with PMHC and 3,5-DHA[a] used separately, and with equimolar mixture of PMHC/3,5-DHA.	**S-7**
**Table S1.**	Kinetic data determined for non-inhibited peroxidation of MeLin in micelles and in liposomes.	**S-4**
**Table S2.**	Kinetic data determined for inhibited peroxidation of MeLin in micelles.	**S-5**
**Table S3.**	Kinetic data determined for inhibited peroxidation of MeLin in liposomes.	**S-6**
**Table S4.**	Kinetic parameters obtained for peroxidation og MeLin in micellar system with PMHC, 3,5-DHA and equimolar mixture of PMHC/3,5-DHA.	**S-8**
**Table S5.**	Comparison of pK_a values for RSV.	**S-8**
	Discussion about pKa for resveratrol	**S-9**
	References	**S-10**

[a] *Abbreviations*: ABAP, 2,2'-azobis(2-methylpropionamidine) dihydrochloride; MeLin, methyl linoleate; PMHC, 2,2,5,7,8-pentamethyl-6-hydrochroman; RSV, resveratrol; 3,5-DHA, 3,5-dihydroxybenzyl alcohol..

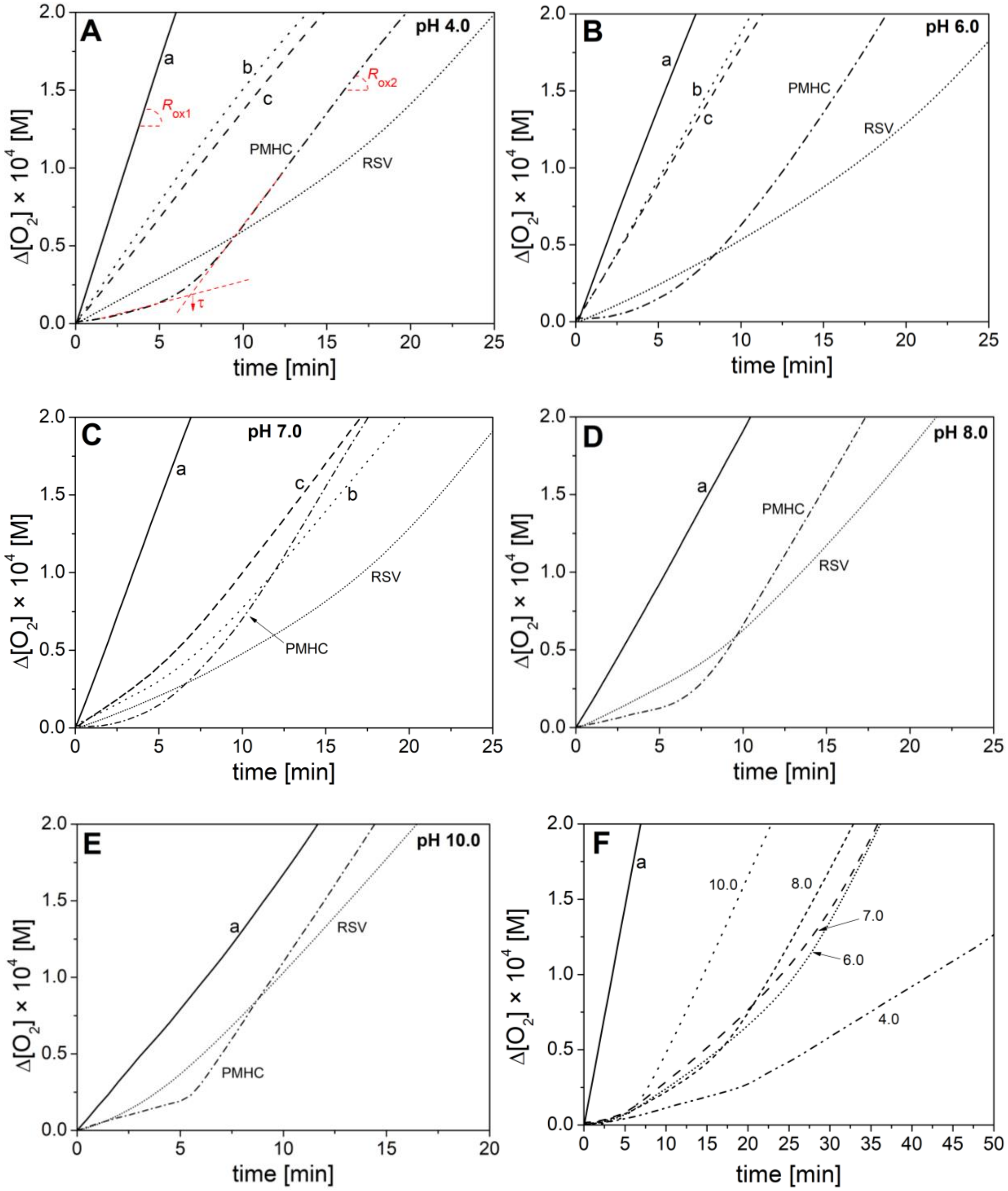

Figure S1. Oxygen uptake for peroxidation of methyl linoleate suspended in Triton X-100 micelles (2.74 mM MeLin, 8 mM Triton X-100) initiated with 10 mM ABAP at 37 °C. In each panel line a denotes spontaneous (non-inhibited) peroxidation, and other lines were recorded for peroxidation in the presence of 1 μM of the following phenols: PMHC, RSV, 3,5-DHA (line b), and 4-HBA (line c). Panels A-E: pH 4, 6, 7, 8, and 10. Panel F: equimolar mixture of PMHC/resveratrol at pH 4.0 – 10.0 (the numbers correspond to pH value).

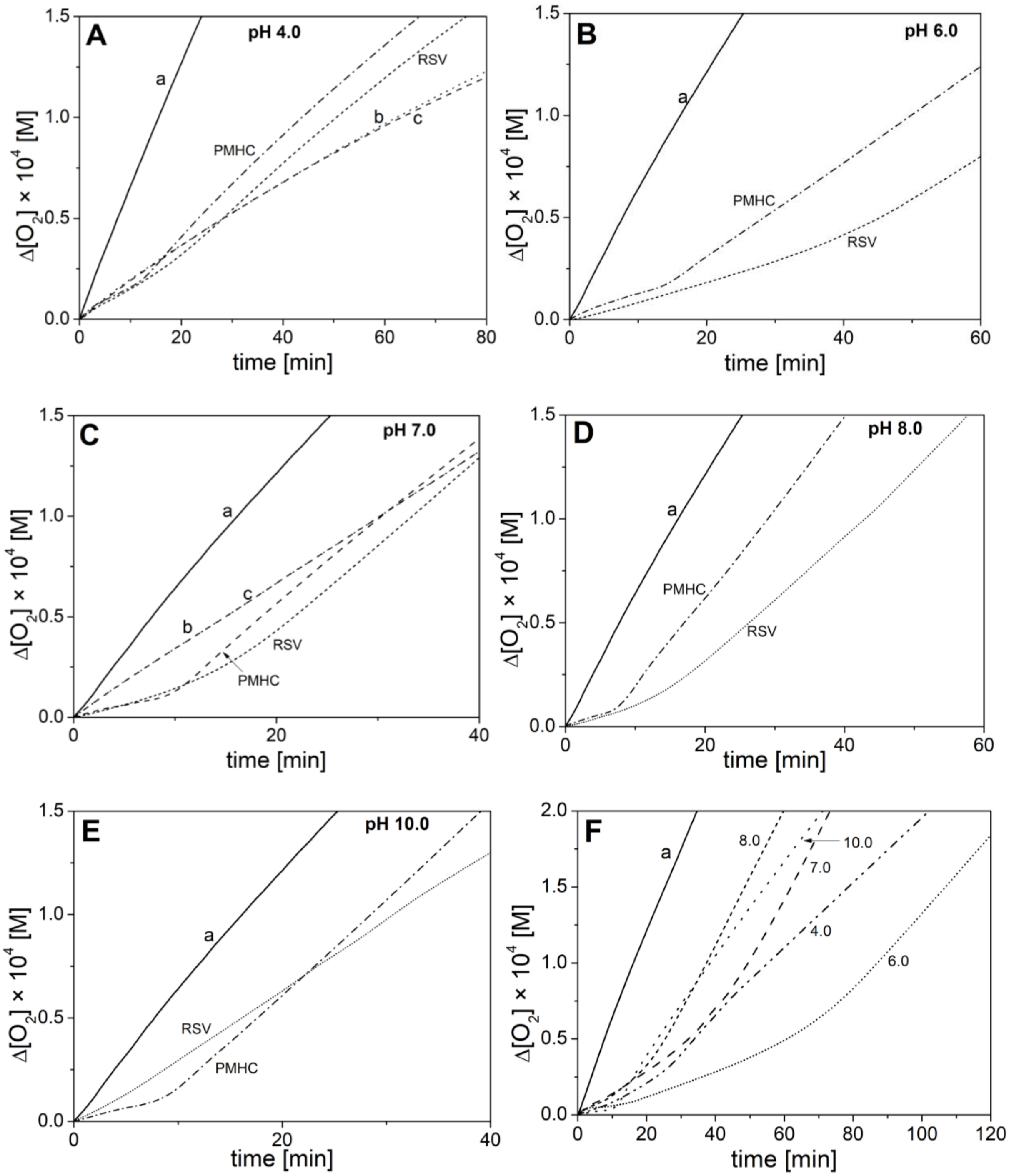

Figure S2. Oxygen uptake for peroxidation of methyl linoleate in DMPC liposomes (2.74 mM MeLin, 20.2 mM DMPC) initiated with 10 mM ABAP at 37 °C. In each panel line a denotes spontaneous (non-inhibited) peroxidation, and other lines were recorded for peroxidation in the presence of 1 μM of the following phenols: PMHC, RSV, 3,5-DHA (line b), and 4-HBA (line c). Panels A-E: pH 4, 6, 7, 8, and 10. Panel F: equimolar mixture of PMHC/resveratrol at pH 4.0 – 10.0 (the numbers correspond to pH value).

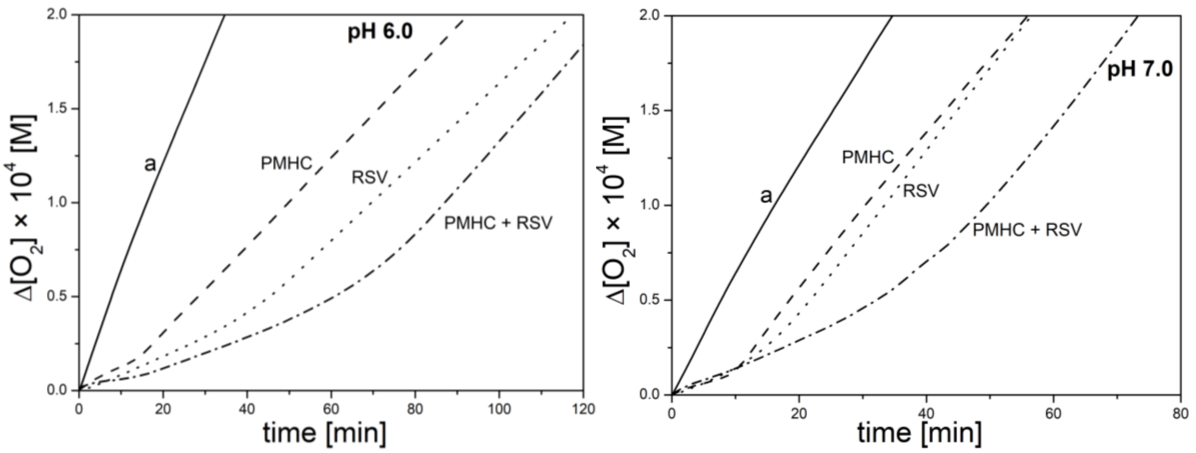

Figure S3. Oxygen uptake for peroxidation of MeLin in DMPC liposomes (2.74 mM MeLin, 20.2 mM DMPC) initiated with ABAP at 37 °C, at pH 6.0 and 7.0: without inhibitor (curve a), with 1 μM of: PMHC, resveratrol and equimolar mixture of PMHC/resveratrol.

Table S1. The rates of initation, R_i, the rates of non-inhibited process, R_{ox1}, kinetic chain lengths, v_{ox1}. R_{ox1} determined for peroxidation of methyl linoleate in micelles and in liposomes at 37 °C at pH 4.0, 6.0, 7.0, 8.0 and 10.0 initiated by 10 mM ABAP and inhibited by 1 μM PMHC.[a]

pH	R_i / nMs^{-1}	$10^8 \times R_{ox}$ / Ms^{-1}	v_{ox1}[b]
**Triton X-100 micelles**[c]			
4.0	4.6 ± 0.2	55.4 ± 3.9	120
6.0	4.7 ± 0.4	47.2 ± 1.0	100
7.0	4.4 ± 0.4	35.7 ± 0.8	81
8.0	4.5 ± 0.3	35.2 ± 4.5	78
10.0	5.7 ± 0.5	27.6 ± 2.1	48
**DMPC liposomes**[d]			
4.0	3.1 ± 0.2	8.4 ± 0.6	27
6.0	2.3 ± 0.1	8.7 ± 0.4	37
7.0	3.8 ± 0.4	9.9 ± 1.7	26
8.0	3.4 ± 0.2	10.0 ± 2.0	29
10.0	3.6 ± 0.2	6.9 ± 0.1	19

[a] Results averaged from at least 6 separate runs. Values were expressed as the mean ± standard deviation (SD).
[b] The kinetic chain length v is the number of peroxidation cycles triggered by one initiating radical. For non-inhibited autoxidation, $v_{ox1} = R_{ox1}/R_i$.
[c] 2.74 mM MeLin in 8 mM Triton X-100.
[d] 2.74 mM MeLin in 20.2 mM DMPC liposomes.

Table S2. Stoichiometric factors *n* calculated for resveratrol, 3,5-dihydroxybenzyl alcohol (3,5-DBA), 4-hydroxybenzyl alcohol (4-HBA) and equimolar mixture of PMHC/resveratrol.. Values R_i were taken from Table S1, for PMHC *n*=2.0. Initial concentration of phenols were always 1.0 μM The experiments were performed in **Triton X-100 micelles** at 37 °C and pH 4.0, 6.0, 7.0, 8.0 and 10.0. Peroxidation of methyl linoleate was initiated by 10 mM ABAP. Values R_i and τ are expressed as the mean ± standard deviation (SD).

ArOH	pH	R_i /nMs^{-1}	τ /min	*n*
PMHC	4.0	4.6 ± 0.2	7.2 ± 0.1	2.0 [a]
	6.0	4.7 ± 0.4	7.2 ± 0.6	2.0 [a]
	7.0	4.4 ± 0.4	7.6 ± 0.7	2.0 [a]
	8.0	4.5 ± 0.3	7.5 ± 0.5	2.0 [a]
	10.0	5.7 ± 0.5	5.8 ± 0.7	2.0 [a]
RSV	4.0	4.6 ± 0.2	15.6 ± 0.8	4.3 [b]
	6.0	4.7 ± 0.4	17.2 ± 0.7	4.9 [b]
	7.0	4.4 ± 0.4	16.2 ± 1.1	4.3 [b]
	8.0	4.5 ± 0.3	11.4 ± 0.2	3.1 [b]
	10.0	5.7 ± 0.5	4.6 ± 0.4	1.6 [b]
3,5-DBA	4.0	4.6 ± 0.2	-	- [c]
	7.0	4.4 ± 0.4	6.8 ± 0.3	1.8 [b]
4-HBA	4.0	4.6 ± 0.2	-	-
	7.0	4.4 ± 0.4	6.4 ± 0.2	1.7 [b]
PMHC/RSV	4.0	4.6 ± 0.2	21.5 ± 0.5	3.9 [d]
	6.0	4.7 ± 0.4	25.7 ± 1.3	5.2 [d]
	7.0	4.4 ± 0.4	23.4 ± 1.7	4.2 [d]
	8.0	4.5 ± 0.3	18.5 ± 0.8	3.0 [d]
	10.0	5.7 ± 0.5	6.6 ± 0.7	0.3 [d]

[a] For Tocopherol and PMHC n = 2.0. [b] calculated from equation 8 (main manuscript) $n = \tau R_i/[\mathrm{ArOH}]_0$. [c] For these systems, the inhibition time was not detected, and n could not be calculated. [d] For PMHC/RSV parameter *n* corresponds to RSV and was calculated from equation: $n_{RSV} = R_i(\tau_{PMHC/RSV} - \tau_{PMHC})/[RSV]$ adapted from general equation proposed by Amorati *et al.*.[1]

Table S3. Stoichiometric factors *n* calculated for resveratrol, 3,5-dihydroxybenzyl alcohol (3,5-DBA), 4-hydroxybenzyl alcohol (4-HBA) and equimolar mixture of PMHC/resveratrol from equation (8) modified to $\tau = n = \tau R_i/[\text{ArOH}]_0$. Values Ri were taken from Table S1, for PMHC n=2.0. Initial concentration of phenols were always 1.0 μM The experiments were performed in **DMPC liposomes (LUVs)** at 37 °C and pH 4.0, 6.0, 7.0, 8.0 and 10.0. Peroxidation of methyl linoleate was initiated by 10 mM ABAP. Values R_i and τ are expressed as the mean ± standard deviation (SD).

ArOH	pH	R_i /nMs^{-1}	τ /min	*n*
**PMHC**	4.0	3.1 ± 0.2	10.9 ± 0.6	2.0 [a]
	6.0	2.3 ± 0.1	14.6 ± 0.5	2.0 [a]
	7.0	3.8 ± 0.4	8.6 ± 0.7	2.0 [a]
	8.0	3.4 ± 0.2	9.8 ± 0.5	2.0 [a]
	10.0	3.6 ± 0.2	9.4 ± 0.7	2.0 [a]
**RSV**	4.0	3.1 ± 0.2	18.1 ± 0.9	3.4
	6.0	2.3 ± 0.1	40.8 ± 1.0	5.6
	7.0	3.8 ± 0.4	14.8 ± 0.6	3.4
	8.0	3.4 ± 0.2	13.6 ± 2.0	2.8
	10.0	3.6 ± 0.2	-	- [b]
**3,5-DBA**	4.0	3.1 ± 0.2	-	- [b]
	7.0	3.8 ± 0.4	-	-
**4-HBA**	4.0	3.1 ± 0.2	-	- [b]
	7.0	3.8 ± 0.4	-	-
**PMHC/RSV**	4.0	3.1 ± 0.2	33.1 ± 4.0	4.1 [c]
	6.0	2.3 ± 0.1	64.4 ± 2.1	6.9
	7.0	3.8 ± 0.4	40.8 ± 1.4	7.3
	8.0	3.4 ± 0.2	21.3 ± 2.8	2.3
	10.0	3.6 ± 0.2	10.8 ± 0.3	0.3

[a] For Tocopherol and PMHC n = 2.0. [b] For these systems, the inhibition time was not detected, and *n* could not be calculated. [c] For PMHC/RSV parameter *n* corresponds to RSV and was calculated from equation: $n_{RSV} = R_i(\tau_{PMHC/RSV} - \tau_{PMHC})/[RSV]$ adapted from general equation proposed by Amorati *et al.*.[1]

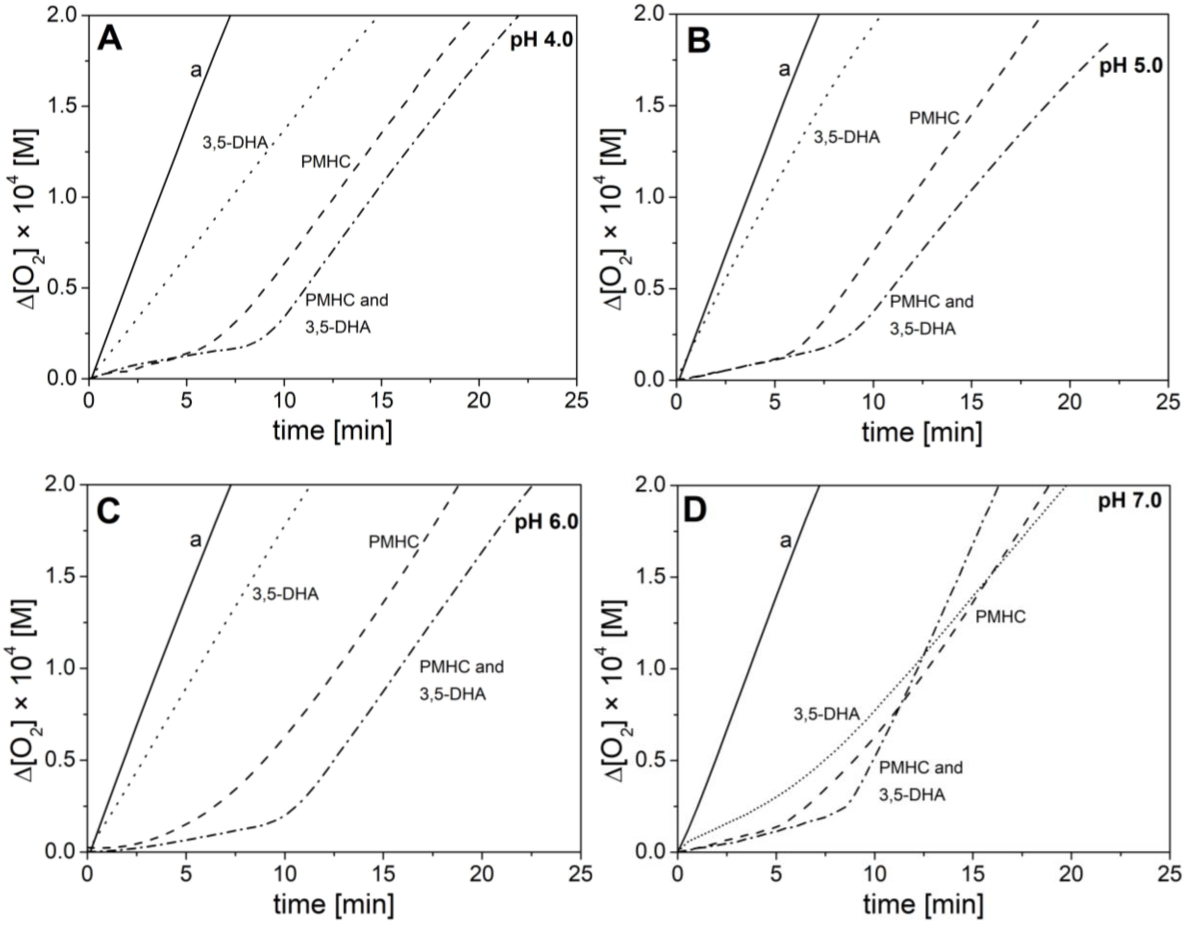

Figure S4. Oxygen uptake for peroxidation of methyl linoleate suspended in Triton X-100 micelles (2.74 mM MeLin, 8 mM Triton X-100) initiated with 10 mM ABAP at 37 °C, at pH 4.0-7.0: without inhibitor (curve a), with 1 μM of: PMHC, 3,5-DHA and equimolar mixture of PMHC/3,5-DHA.The presented results are discussed in the main manuscript text.

Comment to pH 7:
This is the only pH (within the range 4-7) at which 1μM 3,5-DHA is able to inhibit the lipid peroxidation in micellar system, with τ=6.8 ± 0.3 min and the rate of inhibited process R_{inh}=1.09×10^{-7} Ms^{-1}. At the same pH for 1μM PMHC $\tau_{3.5\text{-}DHA}$=7.6 ± 0.7 min and R_{inh}=0.37×10^{-7} Ms^{-1}.When both compounds are used as mixture (1μM each), τ_{PMHC}=9.3 ± 0.7 min and R_{inh}=0.35×10^{-7} Ms^{-1} (the kinetic parameters are listed in Table S4). Because this $\tau_{PMHC/3.5\text{-}DHA}$ is smaller than a sum of induction times of both compounds used separately, there is no additive inhibition. Comparison of R_{inh} parameters demonstrates that PMHC is leading antioxidant, responsible for trapping the peroxyl radicals, but PMHC is only partially recovered by 3,5-DHA.

Table S4. The lengths of induction periods, τ, stoichiometric factors, n, rate of inhibited peroxidation, R_{inh}, the slow-down factors (R_{ox}/R_{inh}, the ratio of the rate of non-inhibited process to the rate of inhibited process), the inhibition rate constants, k_{inh}, and kinetic chain lengths, $v_{inh}=R_{inh}/R_i$ determined for autoxidation of 2.74 mM MeLin dispersed in 8 mM Triton X-100 micelles in the presence of 1 μM of PMHC, 3,5-DHA or an equimolar mixture of PMHC/3,5-DHA. The experiments were performed at 37 °C and pH 4.0, 5.0, 6.0 and 7.0. Peroxidation was initiated by 10 mM ABAP. Values are expressed as the mean ± standard deviation (SD).

pH	τ /min	n	R_{inh} /nMs^{-1}	R_{ox}/R_{inh} [a]	10^{-3} k_{inh} /M^{-1}s^{-1}	v_{inh}
			PMHC			
4.0	7.2 ± 0.1	2.0 [b]	35 ± 1	15.8	10.9 ± 2.2	7.6
5.0	6.4 ± 0.5	2.0 [b]	32 ± 3	15.6	11.6 ± 2.3	5.9
6.0	7.2 ± 0.6	2.0 [b]	52 ± 7	9.1	6.7 ± 1.3	11.1
7.0	7.6 ± 0.7	2.0 [b]	37 ± 5	9.6	18.8 ± 3.8	8.4
			3,5-DHA			
4.0	-	-	231 ± 1 [c]	2.4	- [e]	50.2
5.0	-	-	320 ± 5	1.6	-	59.3
6.0	-	-	304 ± 7	1.6	-	65.7
7.0	6.8 ± 0.3	1.8	109 ± 4	3.3	3.0 ± 0.6	24.8
			PMHC/3.5-DHA			
4.0	8.5 ± 0.6	0.3 [e]	36 ± 8	15.4	- [f]	7.8
5.0	9.0 ± 1.2	0.7	27 ± 4	18.4	-	5.0
6.0	10.3 ± 0.6	0.8	16 ± 3	29.5	-	3.4
7.0	9.3 ± 0.4	0.4	35 ± 8	10.2	-	8.0

[a] The (R_{ox}/R_{inh}) ratio informs how many times the inhibited oxidation is slower than spontaneous (non-inhibited) process (for *Rox* as well as R_i values see Table S1). [b] For α-TOH and PMHC n = 2.0. [c] There was no inhibition period, R_{inh} means rate of retardation. [d] For these systems, the inhibition time was not detected, and k_{inh} could not be calculated. [e] For PMHC/RSV parameter n corresponds to RSV and was calculated from equation: $n_{RSV} = R_i(\tau_{PMHC/RSV} - \tau_{PMHC})/[RSV]$ adapted from ref.[52] [f] k_{inh} cannot be calculated for mixed antioxidants.

Table S5. Comparison of pK_a values for RSV.

pK_{a1} / pK_{a2} / pK_{a3}	Method[a], solvent	Ref.
6.4 / 9.4 / 10.5	S, in H_2O	2,3
6.7 / 9.0 / 11.2	S	4
8.0 / 9.9 / 10.5	S, in H_2O[b]	5
8.2 / 9.7 / -	S in H_2O and SF in H_2O/EtOH (6:4)	6
8.8 / 9.8 / 11.4	SF, H_2O	7
9.0 / 9.8 / 11.3	NMR titration, in H_2O/D_2O (95:5)	8
9.1 / 9.7 / 10.5	S and SF,in H_2O	9
9.3 / 10.0 / 10.6	S, perhaps in H_2O	10
9.5 / - / -	Electrophoresis, in H_2O	11
8.5 / 9.1 / 10.1	QSAR prediction	12
9.7 / 9.6 / 10.6	Theoretical	9
9.2 / - / -	Theoretical	13

[a] S - spectrophotometric titration, SF - spectrofluorimetric titration. [b] Measured in H_2O/MeOH, extrapolated to 100% H_2O. [c] Value predicted by Marvin sketch calculator software from Chemaxont, however, the order of deprotonation indicated one of the resorcinol OH groups (3 or 5) as the most acidic site, followed by deprotonation of OH at position 4'.

Table S5 collects the accessible values of experimentally determined pK_a in water, and two latest parameters are theoretical values. There is a general agreement that 4'-OH group is the most acidic site in RSV (supported by NMR titration[8]), however, the data for first deprotonation are scattered within three pK_a units (!), with two lowest pK_{a1} dramatically different from the others, including theoretical ones. We have to exclude pK_a < 7 because none of the structural fragments in RSV is able to increase the acidity of RSV to be comparable to (or stronger than) the acidity of phenols with strongly electron withdrawing groups: 4-NO_2-phenol (7.15), 4-CN-phenol (7.97), 4-OH-benzaldehyde (7.6) or 4-OH-acetophenone (8.05).[14]

We suppose that such discrepancy between the experimental pK_a values is caused by photo-sensitivity of RSV with possible excitation and isomerization that might happen during spectrophotometric and spectrofluorimetric titrations. Simkovitch and Huppert[15] used the steady-state and time-resolved fluorescence techniques to study E/Z isomerization of RSV and noticed that in the first excited singlet state RSV* becomes much stronger acid than the ground state RSV (pK_a* is several units lower than pK_a) and can transfer a proton to a weak base like, for example, acetate anions. The authors proposed that the excited-state proton-transfer (ESPT) process from RSV* is a form of endogenous defense against fungi, bacteria, and injury, when upon constant exposition to sunlight the steady state pH ca. 4.2 can be generated.[15] The photo-instability of RSV subjected to weak UV irradiation (in standard spectrofluorimeter) was described by Manfredi *et al.*,[4] who reported that successive excitation at 300 nm (at pH 3.6) induced a blue shift in fluorescence emission (250 nm) and *trans*-RSV even after a single excitation was converted into *cis*-RSV intermediate, which, if subjected to next excitations, underwent further photodecomposition to species with carbonyl groups (IR analysis). Photo-rearrangement of stilbene derivatives is acid catalysed,[16] however, when hydroxy group are present in stilbene (as in RSV) no acid is needed and RSV is photo-converted to resveratrone.[17] Zimanyi *et al.*[9] also criticized the pK_a's determined by spectrofluorimetric titration as well as measurements of acidity constants carried out in the presence of oxygen. Additionally, they calculated the energy profile for cascade mechanism of decomposition starting from the electron transfer from RSV anion to molecular oxygen (exothermic process, ~8 kcal/mol), and subsequent (solvent cage) recombination of generated $O_2^{\bullet-}$ with RSV radical having an odd electron at C7 (stilbene site). Dioxetane intermediate was irreversibly cleaved into 4-hydroxybenzaldehyde and 3,5-dihydroxybenzaldehyde anions.[9] Taking into account all above arguments, we disregard the lowest pK_a's from Table 4 as contaminated by pK_a*, and we believe that pK_a ~ 9.0 (determined by NMR titration and close to theoretical one) is the most relevant to acidity of RSV in water. Such relatively high pK_a perfectly supports the localization of a large fraction of RSV in lipid bilayer at pH ≤ 8: partition coefficient determined by Neves *et al.*[12] in LUV (DPMC) at pH 7.4 at temp 37°C is 2797 ± 486 (or log$D$ = 3.43, see Table 1) and should not be dramatically changed at lower pH. Moreover, if pKa was close to 6 or 7, RSV would be very unstable at pH > pKa, that is not the case, because fast degradation of RSV starts at pH > 8 (see discussion of results obtained at pH 10 in the main manuscript).

REFERENCES

(1) Amorati, R.; Ferroni, F.; Lucarini, M.; Pedulli, G. F.; Valgimigli, L. A quantitative approach to the recycling of α-tocopherol by coantioxidants *J. Org. Chem.* **2002**, *67*, 9295-9303.

(2) Stojanović, S.; Brede, O. Elementary reactions of the antioxidant action of trans-stilbene derivatives: resveratrol, pinosylvin and 4-hydroxystilbene *Phys. Chem. Chem. Phys.* **2002**, *4*, 757-764.

(3) Stojanović, S.; Sprinz, H.; Brede, O. Efficiency and Mechanism of the Antioxidant Action of trans-Resveratrol and Its Analogues in the Radical Liposome Oxidation *Arch. Biochem. Biophys.* **2001**, *391*, 79-89.

(4) Manfredi, C.; Trifuoggi, M.; Amoresano, A.; Vasca, E.; Pepe, C.; Volino, S.; Annetta, M. On Trans-Resveratrol in Aqueous Solutions *J. Solution Chem.* **2017**, *46*, 2214-2230.

(5) Takagai, Y.; Kubota, T.; Kobayashi, H.; Tashiro, T.; Takahashi, A.; Igarashi, S. Adsorption and desorption properties of trans-resveratrol on cellulose cotton *Anal. Sci.* **2005**, *21*, 183-186.

(6) Díaz, T. G.; Merás, I. D.; Rodríguez, D. A. Determination of resveratrol in wine by photochemically induced second-derivative fluorescence coupled with liquid–liquid extraction *Anal. Bioanal. Chem.* **2007**, *387*, 1999-2007.

(7) Lopez-Nicolas, M. J.; Garcia-Carmona, F. Aggregation and pKa Values of E-Resveratrol as Determined by Fluorescence Spectroscopy and UV-Visible Absorption *J. Agric. Food Chem.* **2008**, *56*, 7600.

(8) Orgován, G.; Gonda, I.; Noszál, B. Biorelevant physicochemical profiling of (E)-and (Z)-resveratrol determined from isomeric mixtures *J. Pharm. Biomed. Anal.* **2017**, *138*, 322-329.

(9) Zimányi, L.; Thekkan, S.; Eckert, B.; Condren, A. R.; Dmitrenko, O.; Kuhn, L. R.; Alabugin, I. V.; Saltiel, J. Determination of the pKa Values of trans-Resveratrol, a Triphenolic Stilbene, by Singular Value Decomposition. Comparison with Theory *J. Phys. Chem. A* **2020**, *124*, 6294-6302.

(10) Deak, M.; Falk, H. On the chemistry of the resveratrol diastereomers *Monatsh. Chem.* **2003**, *134*, 883-888.

(11) Cao, J.; Chen, G. H.; Du, Y. S.; Hou, F. F.; Tian, Y. L. Determination of Dissociation Constants of Resveratrol and Polydatin by Capillary Zone Electrophoresis *J. Liq. Chromatogr. Relat. Technol.* **2006**, *29*, 1457.

(12) Neves, A. R.; Nunes, C.; Amenitsch, H.; Reis, S. Effects of resveratrol on the structure and fluidity of lipid bilayers: a membrane biophysical study *Soft Matter* **2016**, *12*, 2118-2126.

(13) Cordova-Gomez, M.; Galano, A.; Alvarez-Idaboy, J. R. Piceatannol, a better peroxyl radical scavenger than resveratrol *RSC Adv.* **2013**, *3*, 20209-20218.

(14) (a) Serjeant, E. P.; Dempsey, B. *Ionisation constants of organic acids in aqueous solution*; Pergamon Press, Oxford, 1979; Vol. 23; (b) *CRC handbook of chemistry and physics, 89th edition*; Lide, D. R., Ed.; CRC Press, Taylor and Francis Group: Boca Raton, 2009.

(15) Simkovitch, R.; Huppert, D. Excited-state proton transfer in resveratrol and proposed mechanism for plant resistance to fungal infection *J. Phys. Chem. B* **2015**, *119*, 11684-11694.

(16) Ho, J. H.; Ho, T. I.; Liu, R. S. H. Proton-Assisted Switching of Stilbene Analogues Brought by Direct Irradiation *Org. Lett.* **2001**, *3*, 409.

(17) Yang, I.; Kim, E.; Kang, J.; Han, H.; Sul, S.; Park, S. B.; Kim, S. K. Photochemical Generation of a New, Highly Fluorescent Compound from Non-Fluorescent Resveratrol *Chem. Commun.* **2012**, *48*, 3839-3841.
